# Delirium‐Related Distress of Hospitalised Older Adults Within the Framework of the Theory of Unpleasant Symptoms: A Scoping Review of Qualitative Literature

**DOI:** 10.1111/inm.70309

**Published:** 2026-07-16

**Authors:** Irene Best, Ashley Kuzmik, Patrina Sexton Topper, Marie Boltz, Jennifer Yost

**Affiliations:** ^1^ M. Louise Fitzpatrick College of Nursing, Villanova University Havertown Pennsylvania USA; ^2^ College of Pharmacy, University of Michigan Ann Arbor Michigan USA; ^3^ Ross and Carol Nese College of Nursing, Penn State University University Park Pennsylvania USA

**Keywords:** aged, delirium, hospitalization, psychological distress, qualitative research

## Abstract

Delirium, experienced by up to 50% of hospitalised older adults, continues to be underrecognised, undertreated, and often confused with depression or dementia. Distress is a frequent and impactful symptom experienced in delirium, encompassing the individual's holistic biological or emotional response. Delirium‐related distress has been shown to extend beyond the experience of the patient to include the care partners and nurses who are witnesses to delirium. The essence of delirium‐related distress can be best understood through qualitative methodologies; therefore, this review explored the qualitative literature to summarise what is known about delirium‐related distress in hospitalised older adults, their care partners, and the nurses who care for them. The Arksey and O'Malley methodological framework guided the process of conducting this scoping review, and the main concepts of the population, concept, and context were identified per the Joanna Briggs Institute scoping review methodology. A total of fourteen studies were included in this review, spanning qualitative study designs or mixed‐methods designs that reported qualitative data. The Theory of Unpleasant Symptoms framework was used to categorise the factors that influence delirium‐related distress and the symptom's consequences. This scoping review highlighted aspects of this phenomenon that have been less studied qualitatively, namely, the experiences of care partners and nurses, patients with delirium superimposed on dementia, and potential variations in how delirium‐related distress is experienced over time, from acute care to transitional care to long‐term care.

## 1 Introduction

Delirium has prolonged effects on both patients and their loved ones (American Delirium Society—Why Does Delirium Matter?, n.d.). Delirium, a clinical syndrome that affects cognition, impacts awareness and attention. Further, it presents rapidly and may have a fluctuating course (Alexander and Needham 2023; Williams et al. 2020). Patients experiencing delirium may display inappropriate behaviours, emotional lability, psychomotor disturbances, delusions, perceptual disturbances such as hallucinations or illusions, and sleep disturbances (Inouye et al. 2014). The diagnosis of this multifactorial condition requires careful clinical observation, cognitive screening, and assessment. Delirium manifests due to the interrelationship between the level of patient vulnerability and exposure to precipitating or triggering factors (Inouye et al. 2014). Common triggering factors for the onset of delirium include: serious illness, such as from respiratory or circulatory failure; major surgery; sepsis; metabolic imbalances; psychotropic medications; prolonged immobilization; sleep deprivation; and social isolation (Inouye 1999; Wang et al. 2024). Delirium, highly represented among critically ill, hospitalised patients, increases morbidity and mortality (Inouye et al. 2014).

Older age, defined by the World Health Organization as people aged 60 years and older (World Health Organization 2024), is considered a predisposing factor for delirium, contributing to the high representation of delirium among hospitalised populations. In the United States (U.S.), older adults account for almost half of the inpatient hospital days, and between 14% and 24% of older adults are admitted to hospitals with delirium already present (Inouye 2000; Wu et al. 2025). Up to 50% of adults over the age of 65 in the U.S. will experience delirium during hospitalization, which is associated with increased morbidity and mortality (Inouye 2000; Inouye et al. 2014). As the population continues to age, delirium incidence is expected to increase (Inouye 2000). Nevertheless, delirium continues to be underrecognised, confused with depression or dementia, and undertreated (Inouye et al. 2014; Oh et al. 2017).

The negative sequelae of delirium are primarily described in terms of physical outcomes, such as persistent cognitive impairment, increased risk of pneumonia, pressure injuries, seizures, longer hospital stays, and functional decline (Breitbart et al. 2002; Inouye et al. 2014). However, there is growing interest in the currently under‐evaluated psychological outcomes of delirium, such as distress, anxiety, depression, and post‐traumatic stress disorder (PTSD), resulting from a delirium episode (Williams et al. 2020). In fact, emotional distress was one of the final six core outcomes developed by the leading delirium professional organizations to direct future trials (Rose et al. 2021).

The term distress is used in healthcare to describe physical, emotional, and spiritual conditions (Ridner 2004). A working definition of distress is proposed by Ridner (2004) as a ‘non‐specific, biological or emotional response to a demand or stressor that is harmful to the individual (539)’, and a qualitative meta‐synthesis of delirium‐related distress in intensive care units expanded the definition for delirium‐related distress to include emotional, cognitive, physical, and spiritual distress (Boehm et al. 2021).

This review uses the framework of The Theory of Unpleasant Symptoms (TOUS) to describe and characterise the nature of the symptom experience of delirium‐related distress in the context of hospitalised older adults and their care partners (Lenz et al. 2023). According to the TOUS, symptom influencing factors are either physiologic, psychologic, or situational. Physiologic factors are defined as anatomical/structural, physiologic, genetic, illness‐related, and treatment‐related variables (Lenz et al. 2023, 6). The psychologic factors that influence symptoms include both affective and cognitive variables. These include the patient's mood, affective state during or preceding a symptom, the emotional response to the illness or symptom itself, feelings of uncertainty surrounding the symptom, knowledge about the illness, individual coping skills, spirituality, and social supports (Lenz et al. 2023, 6). Lastly, situational factors describe the patient's social and physical environment. These factors may include access to resources such as healthcare, socioeconomic variables, family and social structure and dynamics, the physical characteristics of the care setting, such as temperature, noise, and light levels, and the cleanliness of air or water (Lenz et al. 2023, 7).

Performance outcomes according to the TOUS are the consequences of symptoms. This was defined broadly to include physical parameters, role performance in personal care and social roles, sexual functioning, and cognitive functional outcomes such as memory, concentration, and problem‐solving (Lenz et al. 2023, 8). Performance factors can be negatively impacted by symptoms, but can also be positive in cases where patients seek additional care support or information, or make behavioural changes.

Understanding the experience of patients with delirium is challenging due to reduced capacity for communication and cognitive changes during the acute phase of delirium. Therefore, the patient's account of their experiences is often reliant on their retrospective recall. Although a limited number of quantitative studies have attempted to measure delirium‐related distress in hospitalised older adults, these approaches cannot fully capture the lived experience of the phenomenon. The authentic meaning of a symptom can only be conveyed by those who have experienced it, making qualitative methodologies especially well‐suited to explore delirium‐related distress (Ayllón Garrido et al. 2007; Martins et al. 2014; Morandi et al. 2015; Moura et al. 2022; Partridge et al. 2019).

Research to date demonstrates that delirium‐related distress in hospitalised older adults is not limited to patients alone (Williams et al. 2020). Family members often describe distress when witnessing a loved one's delirium (Martins et al. 2018), and nurses caring for delirious patients similarly report emotional and psychological strain (Baptista et al. 2023). Capturing these diverse perspectives is critical for a comprehensive understanding of delirium‐related distress. Yet, despite this recognition, the existing qualitative evidence focused on hospitalised older adults has not been synthesised to clarify what is currently known and where knowledge gaps remain. The objective of this scoping review was to examine and map the existing qualitative studies on delirium‐related distress in hospitalised older adults, their care partners, and nurses. The scoping review method was selected to align with the primary objectives of the study: to report on the scope of literature and the types of evidence on the topic, and to identify the key characteristics and factors related to the concept (Munn et al. 2018).

## 2 Methods

This scoping review followed the methodological framework of Arksey and O'Malley (2005). This framework adheres to the following stages: stage 1: identifying the research question, stage 2: identifying relevant studies, stage 3: study selection, stage 4: charting the data, and stage 5: collating, summarising, and reporting the results (Arksey and O'Malley 2005). In adherence with the Arksey and O'Malley framework, quality assessment was not required. Additionally, the updated Joanna Briggs Institute scoping review methodology was used to identify the main concepts of this scoping review (Peters et al. 2020). This scoping review adheres to the Preferred Reporting Items for Systematic Reviews extension for Scoping Reviews (PRISMA‐ScR).

### 2.1 Stage 1: Identifying the Research Question

The primary research question for this scoping review was ‘what is known from the existing qualitative literature about the delirium‐related distress of hospitalised older adults, their care partners, and nurses?’

### 2.2 Stage 2: Identifying Relevant Studies

Initial database searches were performed on July 8, 2025, following consultation with a dedicated research librarian. Included databases were The National Library of Medicine PubMed, CINAHL, APA PsycINFO, Scopus, and Web of Science. Appendix C, Table C1 provides the full search strategies. There was no date restriction applied to this search. To search the grey literature on this topic, two key professional organizations were identified: the Gerontological Society of America (GSA) and the Network for Investigation of Delirium: Unifying Scientists (NIDUS). The published GSA conference abstracts for 2023 and 2024 were searched for the keyword ‘delirium,’ and matches were then screened for title and abstract. The NIDUS website section, ‘Delirium Bibliography,’ was searched for the title ‘delirium‐related distress,’ and those matched records were also screened. A hand search of the reference lists of included studies and relevant review articles was performed, and studies meeting the inclusion criteria were included.

This scoping review considered primary research studies with full‐text availability and in the English language. The primary research studies were any qualitative study designs or mixed‐methods designs that reported qualitative data. Review articles were included for full‐text review to identify relevant citations. Appendix A, Table A1 summarises the inclusion and exclusion criteria.

#### 2.2.1 Population

For the study to be included, participants needed to be older adults who had recovered from an episode of delirium while hospitalised, their care partners, and nurses. Studies that included the perspectives of care partners (including relatives, caregivers, spouses, and adult children) of hospitalised older adults with delirium, as well as nurses or other care providers of older adults with delirium, were also considered for this scoping review.

#### 2.2.2 Concept

The concept of delirium‐related distress is the focus of this scoping review. Studies considered for inclusion described characteristics of delirium‐related distress from the perspective of older adult patients with delirium, or their care partners and nurses who bear witness to the delirium. The term acute confusional state (ACS) was considered to be interchangeable with the term delirium. To qualify for inclusion, studies needed to report on the unpleasant emotional symptoms related to the experience of delirium. Acknowledging that the word distress may not always be used in the thematic coding, studies were included if the theme of distress could be extrapolated from qualitative descriptors such as burden, discomfort, strain of care, fear, anxiety, panic, disturbance, anger, grief, loss of control, mistrust, insecurity, guilt, and shame.

#### 2.2.3 Context

This review included diverse specialty units within the acute care hospital setting, such as orthopaedic units, post‐operative units, general medicine units, and intensive care units (ICU).

Inpatient hospice units or studies in the context of palliative care were not included due to phenomenological differences between the constructs of delirium and terminal delirium.

### 2.3 Stage 3: Study Selection

**FIGURE 1:**
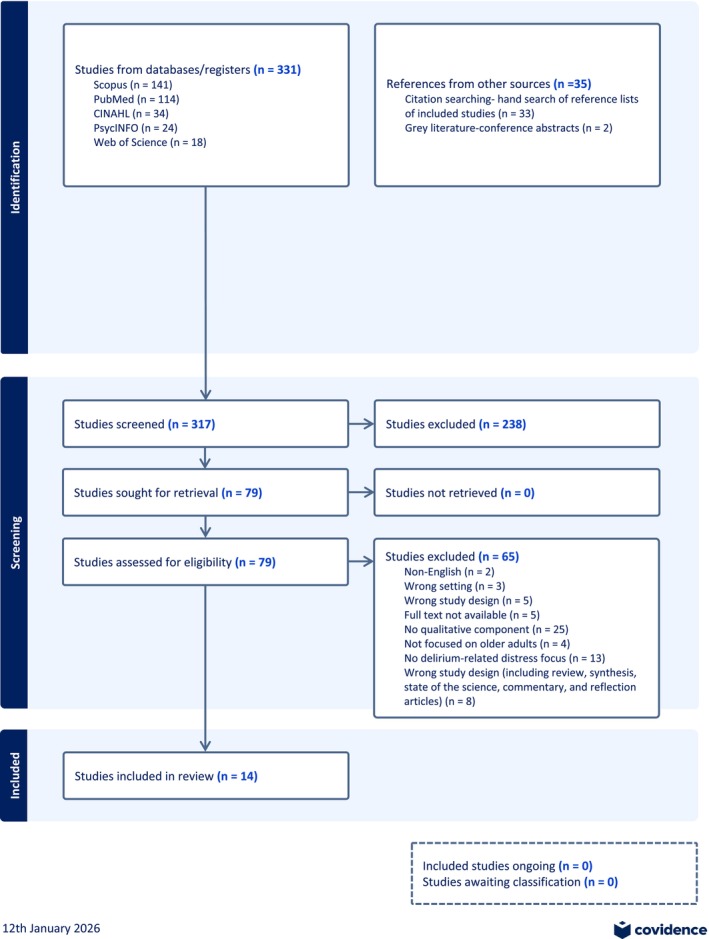
Scoping review delirium‐related distress.

All citations from the database search were uploaded into Zotero Version 6.0.36. From Zotero, the citations were imported into Covidence software Version 2025, where they were duplicated. Two reviewers participated in screening titles and abstracts (IB and AK). Similarly, two reviewers (IB and AK) performed full‐text reviews and discussed any discrepancies until a consensus was reached. The primary reviewer (IB) performed the hand searching of the reference lists of included studies, as well as the grey literature, and uploaded these into Covidence for full‐text review. Study selection was performed using a priori inclusion criteria (Appendix A, Table A1). The search and study selection process is displayed using the Preferred Reporting Items for Systematic Reviews and Meta‐Analyses (PRISMA) flow diagram, which is included as Figure 1.

### 2.4 Data Charting and Extraction

Data were extracted from included studies by the primary reviewer (IB) using the JBI QARI data extraction tool for qualitative research (Peters et al. 2020). A table was constructed to systematically collect and organise relevant data (Table 1). The following characteristics of included studies were displayed: the author(s), study methodology, methods used, description of the phenomena, the geographical origin of the study, the setting, cultural context, participants, data analysis techniques, and authors' conclusions. The second reviewer (AK) then checked the data extraction table for accuracy, and consensus was achieved.

**TABLE 1:** Characteristics of included studies.

Studies	Design data collection methods	Phenomena	Setting	Participants	Data analysis methods	Author conclusions
Andersson et al. (2002)	Qualitative Phenomenology Hermeneutic Narrative interviews	The lived experience of ACS	Sweden Postoperative Orthopaedic Hospital unit	Patients (*n* = 50) Age 67–96	Phenomenological hermeneutic analysis of the text was applied, with a focus on the meaning of being confused. Each patient was interviewed on 1–3 occasions for 50–120 min, and was tape recorded and transcribed. Nonverbal expressions such as silence, laughter, and weeping were included. Authors started with naive reading, then structural analysis, then interpretation of the whole.	What takes place during ACS is probably a combination of a patient's life history, their present situation, and a form of communication concerning their emotional state and inner experiences in this new situation. The main theme of the data was being trapped in incomprehensible experiences and a turmoil of past and present and here and there. Three themes were named and several sub‐themes that described the emotions felt during ACS (embarrassment, being in a borderland, being a victim, not in control, threatened).
Bateman et al. (2016)	Mixed‐Methods Quasi‐experimental Pre‐post design Qualitative surveys	Assess the acceptability and feasibility of a training program for volunteers to provide person‐centered care to patients with dementia and/or delirium	Australia Hospital Rural	QUANT Patients (*n* = 64) Age 65 and over, or aboriginal people 50 and older QUAL Nurses (*n* = 18) Volunteers (*n* = 18)	Nurses and volunteers completed pre‐ and post‐intervention surveys. Surveys included qualitative items and an open‐ended question about their response to the intervention. Consensus between 2 independent reviewers produced five themes.	The person‐centered care volunteer training program for persons with dementia and/or delirium was highly acceptable and positive for staff and volunteers. This study found that this program is feasible to introduce and maintain over time to improve the quality of care of patients with dementia and/or delirium in similar settings. Nurses felt that the volunteers on the units complemented and supported the nursing role.
Sörensen Duppils and Wikblad (2007)	Qualitative Interviews with content analysis/metaphor construction from the content analysis	Describe patients' experience of being delirious	Sweden Postoperative Orthopaedic Hospital unit	Patients (*n* = 15) Age 70–92	Interviews were tape‐recorded and transcribed, and then analysed using content analysis. They lasted from 10 min to just over an hour. The steps of content analysis were described but not specifically referencing an evidence‐based approach. A metaphor based on the categories from the content analysis was used to further describe the phenomenon. The day and night metaphor was used.	Qualitative content analysis yielded three main categories: entry into delirium (change of reality), experiences during delirium (contradictions, dramatic scenes, strong emotional feelings, and difficulties in communicating), and exit from delirium (change back to reality, feelings after delirium, and integration of delirium). The metaphor of day to night was implemented, with twilight as the delirium start, midnight during the delirium experience, daybreak as recovery, and daylight as the reflection on delirium.
Fagerberg and Jönhagen (2002)	Qualitative Phenomenology Interviews	The experience of temporary confusion and the description of the older peoples' reasoning when describing their experience	Sweden Hospital	Patients (*n* = 5) Age 68–90	Based on Husserl's phenomenology, a rigorous step‐by‐step analysis of descriptions of life experiences. Mentions the use of bracketing to avoid the construction of premature explanations. Listed the four steps of the method. Audiotaped interviews, lasting 30–60 min, and then transcribed.	Feeling threat was an experience that all informants shared, and interrelated with this threat was suspiciousness toward others. Another experience shared by all informants was ‘wide open senses,’ meaning they perceived everything happening around them and were unable to concentrate on one specific thing. They desired to escape from the experience. The informant's reasoning and reflection on these emotions focused on feelings of shame and guilt, experiencing humiliation, looking for reasons, and fear of recurrence. There was also a perception by informants that the behaviour of staff could be considered abusive, potentially due to a power imbalance between patients and staff.
la Cour et al. (2024)	Qualitative Multicenter design nested in a randomised controlled trial Interviews and thematic analysis —using a priori themes of delirium experience, delirium‐related stress, and delirium management	The subjective patient experience of delirium, delirium‐related distress, and delirium management in the ICU	Denmark ICU	Patients (*n* = 30) Age 57–85	A structured interview guide with open‐ended questions was developed and pilot tested with 5 patients. This included a description of delirium and common signs of delirium to help them recognise delirium experiences. The DEQ was used in the first section (inductive approach), and then the subsequent section explored specific delirium features inspired by the ICDSC checklist (deductive approach). Two researchers conducted all interviews, and these were audio recorded and transcribed, within 60 days after discharge from ICU when patients were delirium‐free. The transcripts were coded with each of the three themes pre‐determined, using the inductive thematic analysis described by Braun and Clarke. NVivo QRS version 12 supported analysis.	ICU survivors frequently recall the delirium experience as unpleasant and frightening. This leads to delirium‐related distress during and after their ICU stay. The following sub‐themes were reported from the a priori main themes identified. The sub‐themes of delirium experience were: delirium is more vivid than dreams, delirium impacts memory, being at another place or time, and being with unfamiliar people or animals. The sub‐themes related to delirium‐related distress included: mistrust and confusion lead to distress, memory loss leads to distress, and loss of control leads to distress. Finally, delirium management had the sub‐themes of: delirium care fails to alleviate delirium distress, family presence alleviates delirium distress, and physical contact alleviates delirium distress.
Lange et al. (2022)	Qualitative Phenomenology (inspired by Patient Centered Care PCC and Family Centered Care FCC models) Semi‐structured interviews	Patients' and families' experiences of delirium during their ICU stay	Poland ICU	Patients (*n* = 8) Age 61–80 Caregivers (*n* = 8)	Two nurses conducted semi‐structured interviews 1 month after discharge from the hospital using a pre‐designed questionnaire that allowed interviewers to ask additional in‐depth questions. The questionnaire was written in Polish and referred to delirium with the term emotional disorder. Audio recordings were transcribed and checked for discrepancies. Thematic analysis was used, but the approach was not labelled or described.	Six themes were identified during analysis: education, feelings before the delirium (anger, hallucinations), pain and thirst (contributed to aggression and anxiety), the day after (feeling weak and drowsy), talking to the family/patient, and returning home (most families had no concerns). The main problem for both patients and their families was the lack of education about delirium and the need for information in this area. Feelings associated with delirium while in the ICU were often shame and embarrassment about their behaviour.
McCurren and Cronin (2003)	Qualitative Phenomenology Descriptive Interviews	The lived experience of delirium in hospitalised elders	USA Hospital	Patients (*n* = 14) Age 65–88	Interviews took place in hospital rooms if the delirium cleared prior to discharge, or afterwards in their homes if necessary. They were guided by three focusing statements about when the patient noticed a change in the ability to think clearly, any behaviour changes, and what helped during this time. Interviews were tape‐recorded and transcribed, then analysed.	Three clusters of themes resulted from the analysis: being in the confusion event, responding to the confusion, and dealing with the confusion. Being in the confusion included themes of awareness, fuzziness, and altered time and place reality. The themes consistent with responding to the confusion were fear, anxiety, anger, and embarrassment. Dealing with the confusion consisted of attempts to cope with the situation, the family reactions, and the nursing staff interventions.
Meilak et al. (2020)	Qualitative (appears to be descriptive, but this label was not stated) Semi‐structured interviews and thematic analysis	Describe the experience of post‐operative delirium and explore the views of patients and relatives to inform the co‐design of an intervention to minimise distress	UK Postoperative Hospital unit	Patients (*n* = 11) Age 66–81 Relatives (*n* = 12)	Individual semi‐structured interviews were conducted, audio recorded, and transcribed. They took place in the outpatient clinic, and the period following the delirious period varied. Thematic analysis aided by NVivo software was performed. No pre‐existing coding framework was used. The One Sheet of Paper method was used to achieve consensus about themes.	Relatives felt that prior knowledge of delirium would have helped minimise frustration, confusion, and worry about delirium. Both patients and relatives frequently became tearful and described negative and frightening recollections of delirium. The need for emotional support or counselling to minimise delirium‐related distress was suggested. Relatives valued skillful communication strategies by nursing staff and found speaking to patients in a gentle manner to be beneficial. Participants felt that inadequate information provision increased their distress, and specific recommendations are listed on page. 6. The majority of participants wanted a follow‐up after hospitalization to provide further explanation and discuss any cognitive changes noted. A suggested area of improvement was better staff training and enhancing the public awareness of delirium.
Mortensen et al. (2023)	Qualitative Descriptive Interviews	Explore everyday experiences of critically ill patients with delirium from ICU discharge to 1 year follow‐up; focus on quality of life and cognitive function	Denmark ICU	Patients (*n* = 17) Age 57–73	Data were collected at the 1‐year follow‐up to the AID‐ICU trial and collected during home visits. A semi‐structured interview guide was developed, inspired by the RBANS and EQ‐5D‐5L. The interview guide included questions that asked about difficulty remembering things compared to before ICU, any challenges in physical immobility, and if they remember being distressed or confused (from the DEQ). Interviews were digitally recorded and transcribed, lasting 12–28 min. Framework Analysis Method and content analysis were employed in data analysis, and data were organised, and a matrix was developed using NVivo	One main theme was agreed upon: ‘from enduring to adapting.’ The three subthemes were: ‘struggling to regain a functional life,’ ‘struggling to regain normal cognition,’ and ‘distressing manifestations from ICU.’ The most common distressing experience from the ICU was hallucinations. Participants also remembered feeling afraid, alone, and paranoid, distrusting of the families and staff, and feeling embarrassed afterwards. The authors conclude that ICU survivors are often ill‐prepared for the difficulties they will face, and the challenges patients will experience should be communicated to them.
Schofield (1997)	Qualitative Cross sectional design Grounded theory Semi‐structured interviews with constant comparison between transcripts	How older people experienced delirium, if they knew the cause, cure, and whether it left them with feelings of anxiety	England Hospital	Patients (*n* = 19) Aged 66–91	The interviews were stored in written field notes and on tape and transcribed. The primary data were analysed and then related to secondary data obtained from a literature review. Constant comparison of transcripts was used to analyse data.	Core thematic categories were established, namely the nature/quality of the experience (hallucinations, unpleasant memories, wholly unpleasant), reactions to the experience (caution, explanations to relatives, memory loss, reluctance to discuss), and explaining the experiences (little curiosity about the cause or cure of the delirium and quickly dismissed the episode after it passed). The episode of delirium was experienced in the study with bewilderment, surprise, and sometimes curiosity. It was felt that the very process of interviewing patients about their experiences may have been therapeutic.
Schmitt et al. 2019	Qualitative Interpretive description Semi‐structured interviews	Describe the common burden experienced among patients with delirium, their family caregivers, and nurses	USA Hospital	Patients (*n* = 18) Aged 70–92 Family Caregivers (*n* = 16) Nurses (*n* = 15)	Interpretive description was used in the data analysis of the audio‐taped interviews. Atlas.ti was used to manage the data. A very detailed description of coding was performed using two coders for each of the three participant groups. Consensus methodology, including a third coder, was used. A matrix was created to describe the common aspects of delirium burden.	Three common burden themes of the delirium experience: symptom burden, emotional burden, and situational burden. These burdens arise from different sources among patients, family caregivers, and nurses, who have different perspectives on the burden experience. They reinforced that delirium is a shared experience that will respond best to systemwide approaches. All three groups reported that patients' personality changes caused a burden for them. All groups had safety concerns around restraints and experienced burden due to the unpredictability and fluctuating course of delirium.
Stenwall, Sandberg, et al. (2008) (relative study)	Qualitative Descriptive Phenomenology Interviews	To understand the lived experience of close relatives (spouses or adult children, but not a direct caregiver) encountering older persons with ACS	Sweden Hospital	Close relatives (*n* = 10)	Data was collected during the period of ACS and up to 6 months following remission. Interviews, lasting between 35 and 90 min, were tape‐recorded and transcribed. Questions were open and reflective. Interview texts were analysed. A table displayed each analytical step, and an example of how a quotation was analysed in this method.	The essential meaning of an older person with ACS's encounter with a familiar person is that the older person has suddenly become unfamiliar, living in a different reality. This leaves the relatives feeling insecure and limited. The six constituents that illuminate the phenomenon are: change in the other person, rapid and unexpected changes, experiencing insecurity in the encounter, trusting or mistrusting the other person, experiencing loss, and experiencing exposure.
Stenwall, Jönhagen, et al. (2008) (patient study)	Latent Qualitative Content analysis Interviews	To understand the lived experiences of older patients with ACS when encountering professional carers and close relatives	Sweden Hospital (2 geriatric wards)	Patients (*n* = 7) Age 78–98	Patients were interviewed when patients regained lucidity, one week to one month after this point. Interviews lasted 25–65 min. Open‐ended questions were posed about an encounter experienced with a professional carer and an encounter with a close relative. Interviews were audio taped and transcribed, then analysed in 7 steps. A metaphor used in data analysis was a picture of an older patient struggling in a river, not being able to control the situation, but able to control their feelings.	Patients with ACS are searching for answers to what is happening and why. They perceive themselves as hovering between two realities, the ordinary reality and the perceived reality in the ACS. Patients feel dependent on their carers and their willingness to understand and communicate. They may feel lonely, unnecessarily questioned, and untrustworthy, or at times safe, trusted, and understood. Feelings of grief, loss of control, mistrust, insecurity, guilt, and shame were described. Professional carers and close relatives can facilitate trustworthiness by striving toward trusting the older patient, trying to understand the older patient's experiences within the ACS, and giving the patient time to comprehend.
Van Rompaey et al. (2016)	Qualitative Interpretive hermeneutic Semi‐structured interviews	To describe the patient perception of an episode of delirium in the ICU	Belgium ICU	Patients (*n* = 30) All aged adults, but a mean age of 65.2	All interviews were recorded and transcribed, and data analysis was performed with support from NVIVO 8. Interviews were conducted 48–96 h after resolution of delirium. The process involved multiple readings of the text, deriving themes and subthemes. A hermeneutic spiral was used, followed by the interpretation of the researchers.	Data analysis resulted in four major themes: contact and communication, feelings, sleep and time, and implications of the delirious episode. Of the reported feelings, fear was the strongest. Also prominent were frustration and anger, followed by shame and guilt when the delirious episode resolved. The perception of time was disturbed heavily, and this may have contributed to the common problem of sleep disturbance. Patients felt relieved to talk about their experiences, although some felt awkward about recollecting. The patients in this sample were not interested in the cause of the delirium. Also, these patients did not feel suspicious of nurses (as in other studies) and felt their interactions were normal. The start of the delirious period was less remembered, recovery was gradual, and a sense of relief was felt upon the episode's ending.

Abbreviations: ACS, acute confusion syndrome; DEQ, Delirium Experience Questionnaire; EQ‐5D‐5L, The EuroQol 5 Dimension 5 Level; ICDSC, Intensive Care Delirium Screening Checklist; ICU, intensive care unit; RBANS, Repeatable Battery for the; Assessment ofNeuropsychological Status.

The Theory of Unpleasant Symptoms (TOUS) was used as a framework to summarise the results of this scoping review, as well as to identify research gaps in the existing qualitative literature (Arksey and O'Malley 2005; Lenz et al. 2023). Each of the studies' findings was categorised through the lens of the TOUS, noting their interpretations of delirium‐related distress‐influencing and performance factors (Table 2). The three major concepts of the theory are the symptom or symptom cluster, influencing factors, and performance outcomes. Each article was reviewed for its frequency of reporting of data reflecting influencing factors (physiologic, psychologic, and situational) and performance outcomes. These are noted in Table 3.

**TABLE 2:** Study characteristics mapped with Theory of Unpleasant Symptoms (TOUS) framework and concepts.

Study	Geography	Participant perspective			Phenomena	Clinical setting			Dementia status		TOUS influencing factors or performance outcomes			
		Patient perspective	Family perspective	Nurse perspective		General medical	Post‐operative	ICU	Patients with dementia were included	Patients with dementia excluded	Physiologic factors	Psychologic factors	Situational factors	Performance outcomes
Andersson et al. (2002)	Sweden	✓			The lived experience of ACS		✓			Unclear		✓	✓	
Bateman et al. (2016)	Australia			✓	Training volunteers to provide person‐centered care to patients with dementia and/or delirium	✓			✓				✓	✓
Sörensen Duppils and Wikblad (2007)	Sweden	✓			Describe patients' experience of being delirious		✓			✓	✓	✓		✓
Fagerberg and Jönhagen (2002)	Sweden	✓			The experience of temporary confusion and the description of the older people's reasoning when describing their experience	✓				✓	✓	✓	✓	✓
la Cour et al. (2024)	Denmark	✓			The subjective patient experience of delirium, delirium‐related distress, and delirium management in the ICU			✓		Unclear	✓	✓	✓	✓
Lange et al. (2022)	Poland	✓	✓		Patients' and families' experiences of delirium during their ICU stay			✓		✓	✓	✓	✓	
McCurren and Cronin (2003)	USA	✓			The lived experience of delirium in hospitalised elders	✓				✓		✓	✓	
Meilak et al. (2020)	UK	✓	✓		Describe the experience of post‐operative delirium and explore the views of patients and relatives to inform the co‐design of an intervention to minimise distress		✓			Unclear		✓	✓	✓
Mortensen et al. (2023)	Denmark	✓			Explore everyday experiences of critically ill patients with delirium from ICU discharge to 1 year follow‐up; focus on quality of life and cognitive function			✓		Unclear				✓
Schofield (1997)	England	✓			How older people experienced delirium, if they knew the cause, cure, and whether it left them with feelings of anxiety	✓				Unclear	✓	✓	✓	
Schmitt et al. (2019)	USA	✓	✓	✓	Describe the common burden experienced among patients with delirium, their family caregivers, and nurses	✓				✓	✓	✓	✓	✓
Stenwall, Sandberg, et al. (2008) (relative study)	Sweden		✓		To understand the lived experience of close relatives (spouses or adult children, but not a direct caregiver) encountering older persons with ACS	✓				✓		✓		
Stenwall, Jönhagen, et al. (2008) (patient study)	Sweden	✓			To understand the lived experiences of older patients with ACS when encountering professional carers and close relatives	✓				✓	✓	✓		
Van Rompaey et al. (2016)	Belgium	✓			To describe the patient perception of an episode of delirium in the ICU			✓		Unclear	✓	✓	✓	✓

Abbreviations: ACS, acute confusional state; ICU, intensive care unit; TOUS, The Theory of Unpleasant Symptoms.

**TABLE 3:** Application of the Theory of Unpleasant Symptoms (TOUS) Framework.

	Examples of factors that influenced delirium‐related distress
Psychological factors Reported in 12 studies	The level of knowledge about delirium increased distress by way of added anxiety (Lange et al. 2022; Meilak et al. 2020). Relatives' observation of behaviour changes in their loved ones increased distress (Lange et al. 2022; Meilak et al. 2020). When nursing staff scolded, made jokes, rushed the patient, or spoke critically, the patient experienced a heightened emotional response, including feelings of uncertainty (McCurren and Cronin 2003; Meilak et al. 2020; Schofield 1997; Stenwall, Jönhagen, et al. 2008). Communication decreased distress when experienced as calming, validating, and empathetic (Fagerberg and Jönhagen 2002; McCurren and Cronin 2003; Meilak et al. 2020).
Situational factors Reported in 10 studies	The presence of family members promoted clear thinking and improved mood (la Cour et al. 2024; McCurren and Cronin 2003; Meilak et al. 2020; Schofield 1997; Van Rompaey et al. 2016). Physical touch, such as hand holding, alleviated loneliness (la Cour et al. 2024). In the hospital environment, increased distress was associated with the colour of the lighting, the lack of signage on the ward (Meilak et al. 2020), and a lack of a clock (Schofield 1997).
Physiologic factors Reported in 8 studies	Cognitive changes that impacted memory and attention increased distress (Sörensen Duppils and Wikblad 2007), such as when patients did not understand when they were being spoken to, or when nurses posed questions too rapidly, patients' distress heightened (la Cour et al. 2024; Sörensen Duppils and Wikblad 2007; Van Rompaey et al. 2016). Sensory changes were commonly reported as distressing, such as: ◦ increased thirst, cold (Sörensen Duppils and Wikblad 2007) ◦ pain (Lange et al. 2022) ◦ perceptual changes, such as sensory overload (Fagerberg and Jönhagen 2002) ◦ reality orientation, hallucinations, and sleep disturbances (Schmitt et al. 2019; Van Rompaey et al. 2016).

## 3 Results

The search yielded 366 citations. After duplicates were removed, the remaining citations were screened for title and abstract (*n* = 317). After screening, 79 were assessed for eligibility via a full‐text review. A total of 14 studies met the inclusion criteria for this review.

### 3.1 Study Design

Of the 14 included studies, the majority (*n* = 12) employed traditional qualitative research designs. Four studies used a phenomenological methodology (Fagerberg and Jönhagen 2002; Lange et al. 2022; McCurren and Cronin 2003; Stenwall, Jönhagen, et al. 2008). Several of the other studies' methodologies were qualitative descriptive, or just generically identified as qualitative, without an explicitly stated methodology. One study used grounded theory methodology (Schofield 1997) and characterised its design as cross‐sectional. The remaining study employed a mixed‐methods design (Bateman et al. 2016).

### 3.2 Geographical Location

**FIGURE B1:**
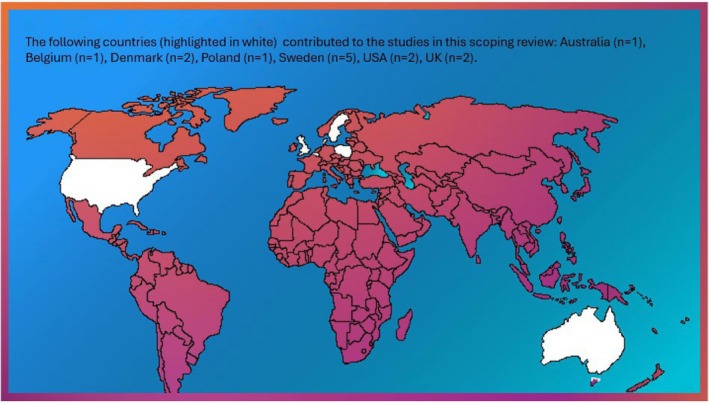
Geographical research matrix of included studies.

Seven countries contributed to the studies included in this scoping review. A graphical representation of the geographical spread of studies can be found in Appendix B, Figure B1. Sweden had the highest number of studies included, with five, followed by the US, England, and Denmark, each with two. The remaining studies were conducted in Australia, Belgium, and Poland.

### 3.3 Clinical Setting

Included in this review were participants who experienced delirium during an inpatient acute care hospitalization or were their relatives or nurses. In most cases, patients were recruited from a general medical ward; however, there were exceptions. Four studies specifically included patients in the ICU (la Cour et al. 2024; Lange et al. 2022; Mortensen et al. 2023; Van Rompaey et al. 2016). Three studies examined patients in the context of postoperative delirium and likewise included participants on an orthopaedic unit (Andersson et al. 2002; Sörensen Duppils and Wikblad 2007) and other non‐specific postoperative units (Meilak et al. 2020). In their study of patients with acute confusion, Stenwall, Jönhagen, et al. (2008) included patients who were admitted to two specific geriatric wards.

### 3.4 Focus of the Studies

#### 3.4.1 Participant Perspectives

This scoping review of the qualitative literature includes studies that describe the phenomenon of delirium‐related distress in hospitalised older adults. The perspectives of patients, care partners, and nurses were considered. Across the literature included in this review, researchers consistently presented the patient perspective (Andersson et al. 2002; Fagerberg and Jönhagen 2002; la Cour et al. 2024; McCurren and Cronin 2003; Mortensen et al. 2023; Schofield 1997; Sörensen Duppils and Wikblad 2007; Stenwall, Sandberg, et al. 2008; Van Rompaey et al. 2016). More than half of the studies (*n* = 9) exclusively described the experience of older adults with delirium.

Two studies included both the perspective of the patient and the family (Lange et al. 2022; Meilak et al. 2020). One study focused on the perspective of professional or volunteer care providers (Bateman et al. 2016). Likewise, one study focused exclusively on the perspective of the close relatives of patients with delirium (Stenwall, Sandberg, et al. 2008). Finally, only one study included all three perspectives in its inquiry: the patient, family caregiver, and the nurse (Schmitt et al. 2019). Based on this summary of participant perspectives, the older‐aged, hospitalised patient's perspective on delirium has been well described. There appears to be a gap in qualitative research from the perspectives of care partners and providers, including familial relations, professional nursing staff, and volunteer staff.

#### 3.4.2 Terminology

Although the purpose of this review was to investigate qualitative studies describing or exploring the phenomenon of delirium‐related distress, the word ‘distress’ was not explicitly stated in the purpose, objectives, aims, or interview guides in most of the studies. Only three studies used the specific terminology of delirium‐related distress (la Cour et al. 2024; Meilak et al. 2020; Mortensen et al. 2023). la Cour et al. (2024) identified an a priori theme of *delirium‐related distress*, and in the study by Meilak et al. (2020), the term *distress* appears in both the study title and aim. Mortensen et al. (2023) included a question about their memory of being distressed or confused in the interview guide.

The majority of studies sought to describe the lived experience of delirium, which invited an authentic narrative account of the episode from the participant, without stated assumptions. Consistent with this approach, the research questions did not presuppose that distress was inherent in the delirium experience for every participant. The concept of distress was therefore introduced through the data in most of the studies and was conceptualised using a variety of descriptors, as noted previously. One such descriptor was delirium burden, which was investigated by Schmitt et al. (2019).

Interestingly, there was no uniformity in the language used to define delirium‐related distress. Studies in this review were split into using the terminology of acute confusional state (ACS) (Andersson et al. 2002; Stenwall, Jönhagen, et al. 2008; Stenwall, Sandberg, et al. 2008), temporary confusion (Fagerberg and Jönhagen 2002), acute confusion, or confusion episode, or the term delirium (Bateman et al. 2016; Meilak et al. 2020; Mortensen et al. 2023; Sörensen Duppils and Wikblad 2007; Van Rompaey et al. 2016), or both confusion and delirium interchangeably, depending on the context (McCurren and Cronin 2003; Schmitt et al. 2019; Schofield 1997). In one study, when speaking to patients and families, the researchers referred to the episode of delirium as the ‘hard time,’ or the ‘emotional disorder’ (Lange et al. 2022). This ambiguity in language may contribute to a lack of conceptual understanding of delirium and associated distress.

The definition of ‘older adult’ varied among the included studies. The majority of studies defined older adults as age 65 or older; however, two studies included participants as young as age 57 (la Cour et al. 2024; Mortensen et al. 2023). Neither Lange et al. (2022), nor Meilak et al. (2020) set out to exclusively include older adults; however, the sample reflected adults aged 61 to 80 and 66 to 88, respectively. One study recruited and included adults of all ages; however, with a mean patient age of 65.2 and meeting all other inclusion criteria, it was included (Van Rompaey et al. 2016).

#### 3.4.3 Inclusion of Dementia

Older adults with dementia or Alzheimer's disease who are hospitalised and experience delirium superimposed on their dementia were not well represented in the included studies. Half of the studies in this review excluded patients with dementia (Fagerberg and Jönhagen 2002; Lange et al. 2022; McCurren and Cronin 2003; Schmitt et al. 2019; Sörensen Duppils and Wikblad 2007; Stenwall, Jönhagen, et al. 2008; Stenwall, Sandberg, et al. 2008). Of the remaining studies, in most cases, it was unclear if patients with dementia were included, as it was not stated directly (Andersson et al. 2002; la Cour et al. 2024; Meilak et al. 2020; Mortensen et al. 2023; Schofield 1997; Van Rompaey et al. 2016). One study did include patients with dementia; however, the study intervention to minimise distress did not differentiate between dementia and delirium (Bateman et al. 2016).

#### 3.4.4 Application of the TOUS Framework

Using the TOUS framework, the most reported influencing factors were psychologic, with 12 of the 14 studies providing evidence of psychological factors influencing delirium‐related distress. Situational factors were reported in 10 of the 14 articles. This category provided the most examples of factors that minimised delirium‐related distress. Physiologic influencing factors, rooted in the physical and condition‐defining characteristics of delirium, were reported in eight out of fourteen studies. Table 3 provides examples of how each type of influencing factor demonstrated an influence on delirium‐related distress.

Mortensen et al. (2023) provided the most data concerning performance outcomes, which was also the only study to provide a longitudinal assessment of delirium outcomes at one year post‐ICU discharge. Their reporting suggested that the greatest challenge followed recovery from delirium was regaining functional capacity. In their sample of participants, none returned to the same level of functional capacity as before their critical illness; many reported needing more rehabilitation and expressed the need to make adaptations physically and cognitively (Mortensen et al. 2023). Some patients still remembered the hallucinations or delusions one year later and remembered their feelings of fear, loneliness, and paranoia (Mortensen et al. 2023). Thinking about the delirium episode after it resolved was reported elsewhere, where patients sought meaning and had a variety of feelings about it, such as fear, dissociation, remorse, humiliation, and relief (Fagerberg and Jönhagen 2002; Sörensen Duppils and Wikblad 2007; Van Rompaey et al. 2016).

## 4 Discussion

The objective of this scoping review was to examine and map the existing qualitative studies on delirium‐related distress in hospitalised older adults, their care partners, and nurses.

This review confirms that delirium‐related distress does occur as part of the lived experience of delirium for hospitalised older adults, and is supported by the personal accounts of patients, care partners, professional nurses, and volunteer care providers. The patient perspective has been the most frequently investigated, and data have produced some similar themes across multiple studies. The identified gaps in the literature are important findings that will provide direction for further study of this phenomenon. These findings are reported categorically by the population, concepts, and context framework of the Joanna Briggs Institute (JBI) (Peters et al. 2020).

### 4.1 Population: Limited Focus on Care Partner Perspective, Nurse Perspective, and Patients With Dementia

Care partners of the patient with delirium, described in this study as family members, relatives, and caregivers, have received limited attention in the scientific literature. Only four studies provided this perspective when considering delirium‐related distress. This gap is noteworthy due to the heavy reliance on family members to provide emotional, cognitive, safety, mobility, and social support to their hospitalised care partner. The presence of family was repeatedly cited as an important influencing factor in relieving distress (la Cour et al. 2024; McCurren and Cronin 2003; Schofield 1997; Van Rompaey et al. 2016). However, care partners found it distressing to observe unfamiliar and unpleasant behavioural changes in patients (Lange et al. 2022; Meilak et al. 2020) and when they struggled to communicate and make connections with their care partner (Schmitt et al. 2019; Stenwall, Sandberg, et al. 2008). This remains an important area for continued exploration due to the critical need for targeted interventions. Specific interventions might address knowledge deficits about delirium and management strategies that care partners vocalised (Meilak et al. 2020; Stenwall, Sandberg, et al. 2008).

Only two studies included nurses' perspectives (Bateman et al. 2016; Schmitt et al. 2019), and no studies focused on professional staff perspectives. Because nursing interventions are critical in the detection and management of patients with delirium, they are an important group to qualitatively study to gain insights into their experience of delirium‐related distress. As noted previously, communication strategies and styles, along with information sharing frequency among professional staff, have a strong impact on how patients perceive and experience delirium.

It was noted that older adults with dementia or Alzheimer's disease, who experience delirium while hospitalised, are underrepresented in this scoping review. This is an important gap to highlight. Individuals with dementia are at a higher risk of experiencing delirium while hospitalised than those without any baseline cognitive impairment (Kolanowski et al. 2010). Dementia is a risk factor in the development of delirium; therefore, it adds to the frequent occurrence of hospitalised older adults with delirium superimposed on dementia (Inouye et al. 2014; Kolanowski et al. 2010). There are challenges in assessing delirium‐related distress in this subpopulation due to communication differences and the need to incorporate observational assessment beyond the standard interview method. It is an area ripe for further investigation.

### 4.2 Concept: Ambiguity in the Conceptual Understanding of Delirium and Delirium‐Related Distress

The term acute confusional state (ACS) was common in the earlier published literature (Andersson et al. 2002; Fagerberg and Jönhagen 2002; McCurren and Cronin 2003; Schofield 1997; Stenwall, Jönhagen, et al. 2008; Stenwall, Sandberg, et al. 2008) as opposed to the current practice of use of the term delirium. These terms were used to describe the same phenomenon, with nomenclature reflective of the trends of the time. In terms of promoting better societal recognition of delirium and a desire to raise public health awareness of delirium, clinicians should be encouraged to use this terminology consistently and to educate patients and families about it, using the clinical term delirium. As mentioned, several studies in this review replaced the word delirium with other descriptors (e.g., confusion episode, hard time, acute confusion, and emotional disorder) when interviewing participants, which risks the precision and therefore the quality of the data.

The most pressing conceptual challenge was the lack of a clear, cohesive definition of delirium‐related distress across researchers. ‘Distress’ was not a consistently used descriptor of the phenomenon, particularly in earlier published studies. The unpleasant emotions and somatic associations that patients and others experienced concerning delirium were not uniformly categorised or named as distress. Instead, a variety of other words attempted to describe the various aspects of their experiences, which contributed to some conceptual ambiguity. For example, Schmitt et al. (2019) use the term delirium burden; however, it is not clear how burden is differentiated from the concept of distress conceptually.

### 4.3 Context: Lack of Temporal Specificity and Longitudinal Data Collection

The qualitative literature describing the experience of delirium during hospitalization provided a variety of geographical and clinical setting contexts. What emerges from this review is a striking lack of understanding of the transitional period following acute hospitalization. Specifically, it remains unclear whether delirium‐related distress persists beyond the acute phase, what factors shape its course during this transition, and which influences worsen or alleviate distress.

The data collection timeframe for the included studies was often wide and variable, e.g., Stenwall et al. (2008) collected data from relatives up to six months following delirium remission, and up to one month from patients. Lange et al. (2022) conducted patient and caregiver interviews one month after hospital discharge, and la Cour et al. (2024) performed their inquiry within 60 days of ICU discharge. Mortensen et al. (2023) was the only study to perform data collection at one year following the delirium episode. Future studies would increase the comprehensiveness of the body of research by tightening the window of data collection. This would provide a better sense of the similarities and differences in how delirium‐related distress is experienced acutely, immediately after resolution of delirium, at the discharge transition point, and then at subsequent timepoints longitudinally.

Delirium‐related distress is a frequent and impactful symptom affecting hospitalised older adults, their care partners, and nurses. Prior research has quantified the levels of distress using measurement tools such as The Delirium Experience Questionnaire (DEQ) (Breitbart et al. 2002). However, the qualitative data from this review add a richer and more vivid elucidation. This scoping review highlighted the aspects of this phenomenon that have been less studied qualitatively, namely, the care partner and nurse perspectives, the perspective of patients with delirium superimposed on dementia, and the potential variations in how delirium‐related distress is experienced over time, from acute care to transitional care to long‐term.

This review included different clinical settings within the acute care hospital setting, representing general medical and surgical units, dedicated orthopaedic units, and ICU. Studies set in the ICU (la Cour et al. 2024; Lange et al. 2022; Mortensen et al. 2023; Van Rompaey et al. 2016) uncovered specific influencing factors that did not pertain to general hospital units. For example, the use of tracheal intubation tubes and face masks amplified the challenges in communication experienced in delirium (la Cour et al. 2024). Additionally, the sensory stimulation of the ICU environment contributed to more difficulty falling asleep (Van Rompaey et al. 2016). Mortensen et al. (2023) studied patients one year following delirium in the ICU and found that functional decline was a significant struggle. It must be noted that this sample experienced a critical illness that contributed to a need for lengthy rehabilitation beyond the effects of the delirium alone. The higher acuity of patient care in the ICU and the corresponding severity of illness of patients may demonstrate some unique factors that influence delirium‐related distress; however, further research is needed to explore this assumption.

This review found that talking about the delirium episode, a frequently cited psychological influencing factor, was described as both helping relieve delirium‐related distress in some participants while adding to delirium‐related distress for others (Andersson et al. 2002; Lange et al. 2022; Schofield 1997; Van Rompaey et al. 2016). It seems to be a therapeutic and valuable intervention for some (Sörensen Duppils and Wikblad 2007), but others found that it conjured feelings of shame (Lange et al. 2022) or disturbed feelings (Schofield 1997). McCurren and Cronin (2003) suggested providing patients with the invitation to discuss their episode of confusion for the sake of those who find the debriefing helpful to find meaning in their experience.

This review focused on qualitative data describing delirium‐related distress in hospitalised older adults and did not include intervention studies aimed at distress reduction specifically. However, there were two examples of delirium management programs described in the included studies that are worth mentioning. Schmitt et al. (2019) referenced the Hospital Life Elder Program (HELP) as a successful strategy for both prevention and treatment of delirium symptoms, inclusive of patients, families, and nurses (Reuben et al. 2000). Some of the interventions included in this program are: therapeutic activity, relaxation therapy, sleep enhancement, social work, chaplaincy consultation, supportive care to families through bedside training, and support for nurses through educational and delirium training. Bateman et al. (2016) created an innovative program that included some of these key elements, providing bedside volunteers trained in feeding, monitoring, and comforting patients in their small, rural hospital setting. This program was found to be acceptable and feasible to both nursing staff and volunteers in their pilot study (Bateman et al. 2016). These examples illustrate efforts to consider the inclusion of delirium‐related distress in delirium management programs; however, more targeted interventions are still needed to address this symptom.

The TOUS was a useful framework to highlight some of the critical influencing factors and to identify the frequencies of the reported evidence of each of the conceptual domains: physiologic, psychologic, and situational. Further research is needed to examine these factors for those that are modifiable, to target specific interventions. Also, future research is warranted to explore the performance outcomes of each of the participants' consequences to delirium‐related distress, both acutely and over time. Mortensen et al. (2023) have given us a glimpse into the potential long‐lasting effects of delirium‐related distress.

### 4.4 Strengths and Limitations

This scoping review followed the well‐defined methodological framework of Arksey and O'Malley (2005) and benefited from the rigorous two‐reviewer process to produce trustworthy results. Use of the JBI Checklist tool (Lockwood et al. 2015) for data extraction enhanced the clarity in cohesively presenting results. Additionally, the use of the TOUS framework was a novel way to summarise factors that influence and are consequences of delirium‐related distress and presented suggestions for actionable improvements in delirium management. To our knowledge, no other review synthesising qualitative research within this population, concept, and context exists. The authors acknowledge that there may be additional articles in the literature that were inadvertently missed in screening that would meet the inclusion criteria. This scoping review contributed to an important first step in determining that a more focused systematic review can be done. A systematic review would answer a precise clinically meaningful question, test the effectiveness of a treatment or intervention, or provide evidence to inform practice (Munn et al. 2018).

A strength of this review is that by providing the perspectives of patients, care partners, and nurses, a comprehensive summary of the qualitative studies on this topic is displayed. Since delirium‐related distress is known to extend beyond just the patient with delirium, the authors were keen to include the other participant perspectives for a more holistic view of the phenomenon. However, it must be acknowledged that results are diluted if one unique participant perspective is isolated due to the limited number of studies in total that met the inclusion criteria for this scoping review.

Similarly, the diverse clinical settings included can be seen as a strength and a limitation. Because there appeared to be similar results reported across the hospital settings, it proved to be a useful strategy to include all acute care units to widen the net of available studies to be considered. One caveat, as noted previously, there may be disparities between general medical and surgical units and the ICU; however, that claim is not sufficiently supported due to the small number of studies included.

Although this review did not critically appraise the quality of the included studies, it was apparent through this review that there was no uniformity regarding delirium screening to validate the presence and/or the resolution of a delirium episode. The included studies collected data at different time points after delirium, which risked the authenticity of the data, presenting a limitation of this study. This approach assumes that the recall of events within days of a delirium episode is comparable to the recall of events weeks or months afterward.

### 4.5 Relevance for Clinical Practice

This scoping review highlighted the time, care, and attention that are required to fully recover from delirium, often coinciding with a critical illness. Once discharged from an acute care hospitalization, there is still significant recovery needed. Participants in these studies expressed a need for emotional, physical, psychological, and cognitive recovery, and rarely is there a structured approach to support patients and care partners in any of these. The promotion of function‐focused care during hospitalization, to counteract the common experience of functional decline in this population, should be prioritised in delirium prevention and management programs (Boltz et al. 2018). Programs such as HELP and other Age‐Friendly care initiatives are effective in delirium prevention, but are not implemented universally (Kwak et al. 2024; Reuben et al. 2000). Bateman et al. (2016) provided an example of a more grassroots effort that can be transferable to other lower‐resourced settings that may not have the capacity to incorporate more sophisticated programs.

Conceptual clarity is important as a primary step to assess, measure, or modulate a phenomenon. Apparent from the results of this review is a lack of consensus on the language and definition used to describe the phenomenon of delirium‐related distress. This ambiguity intensifies the challenges that exist in the implementation of efforts to prevent or reduce delirium‐related distress.

The data excerpts reflected in this review were noteworthy. These personal accounts give insight into the fuzzy perception, muddled thinking, lack of control, and profound loneliness experienced by patients while acutely delirious. Delirium is somatic and visceral. Clearly, patients experience an array of unpleasant emotions during and after delirium. Reading the narrative accounts of participants, which were reported in these qualitative study interviews, evokes an ethical and moral responsibility to respond. Results underscore the need for nurses to investigate effective interventions for the relief of this distress. Key strategies suggested by patients and care partners in this review include listening, being present, validating feelings, recognising the importance of words, and offering small gestures, such as hand holding, which can have a meaningful impact.

In conclusion, the authors are confident that this topic lends itself to a full systematic review of the literature. A systematic review would provide the opportunity to synthesise the existing evidence about delirium‐related distress both quantitatively and qualitatively, and would allow for a deeper investigation into the TOUS concepts. This inquiry would set the stage for intervention studies targeting modifiable factors of delirium‐related distress.

## 5 Conclusion

This scoping review provided a comprehensive overview of studies describing or exploring the symptom of delirium‐related distress, mapping what has and has not yet been studied. The results captured delirium's profound impact on the well‐being of hospitalised older adults, their care partners, and nurses. Through the lens of the TOUS framework, the qualitative literature was explored for the reporting of factors that appeared to influence the experience of delirium‐related distress and the consequences of this symptom. Examining studies from the perspectives of patients, care partners, and nurses reveals the wide‐ranging impact of the phenomenon while also emphasising the unique insights contributed by each group.

The main gaps in the literature identified from this review were: a limited focus on the care partner perspective, the nurse perspective, and persons living with dementia, conceptual ambiguity in the understanding of delirium and delirium‐related distress, a lack of temporal specificity of distress measurement, and a need for longitudinal data collection. It is recommended that future research prioritise the study of these populations and content areas.

## Funding

The authors have nothing to report.

## Disclosure

Authorship Statement: All authors have met the authorship criteria according to the latest guidelines of the International Committee of Medical Journal Editors. All authors are in agreement with the manuscript.

## Conflicts of Interest

The authors declare no conflicts of interest.

## Data Availability

Data sharing is not applicable to this article as no datasets were generated or analysed during the current study.

## Appendix A

**TABLE A1:** Inclusion and exclusion criteria.

Inclusion criteria	Exclusion criteria
Participants	
Older adults age 65 and older	Paediatrics or all aged adults
Patient, care partner, and/or clinician perspective	
Context	
Acute care hospitals	Outpatient settings, including palliative care, hospice
Any country	
Concept	
Delirium‐related distress	
Study characteristics	
Any qualitative or mixed‐methods study design	Any quantitative design, commentary, state‐of‐the‐science, reviews (e.g., narrative, systematic)
Grey literature (i.e., dissertations/theses, conference abstracts)	Not published in English
Published in English	Full‐text unavailable

## Appendix C

**TABLE C1:** Database search from incept to July 08, 2025.

APA PsycINFO^a^	Subject (delirium) AND subject (psychological distress)
CINAHL	MW delirium AND SU psychological distress
The National Library of Medicine PubMed	Delirium[MeSH Terms] AND distress OR delirium[MeSH Terms] AND stress, psychological[MeSH Terms]
Scopus	Keywords delirium AND psychological distress
Web of Science	TS = “delirium‐related distress”

Abbreviations: APA, American Psychological Association; MeSH, medical subject heading; MW, word in subject heading; SU, subject; TS, topic search.

^a^ Included dissertations via ProQuest.
